# “Superman” Latissimus Dorsi Flap for Breast Reconstruction: A Technical Innovation for Covering Extensive Mastectomy Defect

**DOI:** 10.7759/cureus.21514

**Published:** 2022-01-23

**Authors:** Tarush Gupta, Mayank Mangal, Jerry R John

**Affiliations:** 1 Department of Plastic Surgery, Postgraduate Institute of Medical Education and Research, Chandigarh, IND

**Keywords:** reconstructive breast surgery, pedicled latissimus dorsi myocutaneous flap, post mastectomy defect, latissimus dorsi flap, autologous breast reconstruction

## Abstract

Breast reconstruction in extensive post-mastectomy defects is challenging for a reconstructive surgeon. While a plethora of options is available for breast reconstruction, pedicled latissimus dorsi (LD) flap remains the flap of choice for most surgeons. However, the size of the skin paddle of the LD flap may not suffice for extensive defects. We present a technical modification in the planning of the LD flap for its use in extensive defects.

## Introduction

Breast reconstruction is an integral part of breast cancer surgery, aimed at correcting the chest wall defect and symmetry. Over the years, various techniques have been implemented for the reconstruction of the breast: latissimus dorsi (LD) flap, transverse rectus abdominis myocutaneous (TRAM) flap, deep inferior epigastric artery perforator (DIEP) flap, implant, various locoregional, and microvascular free flaps [1]. However, the method of reconstruction is best individualized according to surgical feasibility, patient’s need, and preference [2].

Extensive post-mastectomy defects, however, pose a great challenge to the reconstructive surgeon. It may require a single large free/pedicled flap or a combination of free/pedicled flaps to resurface the extensive defect, which may result in higher donor site morbidity and other complications [3,4]. In extensive defects, the availability of the required size and the volume of the skin paddle may also be an issue. 

Various modifications in the design of harvested LD flap skin paddles have been proposed in the literature to overcome the limitation of the size of the skin paddle [5-7]. We report a case of carcinoma breast with an extensive post-mastectomy defect, resurfaced with a pedicled LD myocutaneous flap with an S-shaped skin paddle, allowing primary closure of the donor site.

## Case presentation

**Figure 1:**
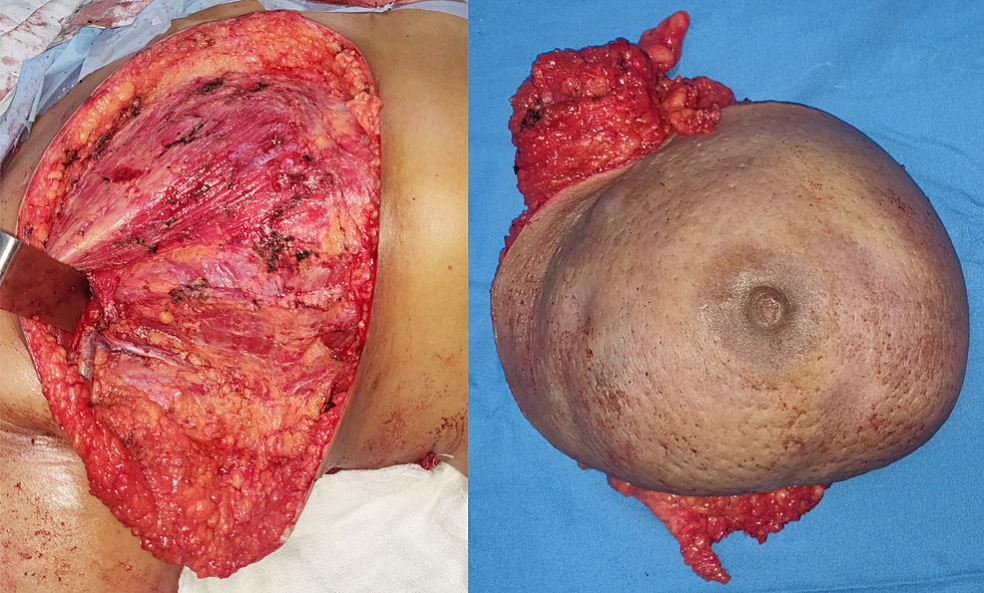
Intraoperative post-mastectomy defect (left), and the excised right breast specimen (right)

We report a case of a 35-year-old patient with carcinoma of the right breast (T4dN1M0), who underwent right modified radical mastectomy with axillary lymph node dissection. The resultant defect is 20 x 16 cm in size (Figure 1).

The patient was scheduled for surgery during the peak COVID-19 crisis, and she did not consent for an extensive reconstruction involving abdominal flaps, so a pedicled LD flap was planned.

**Figure 2:**
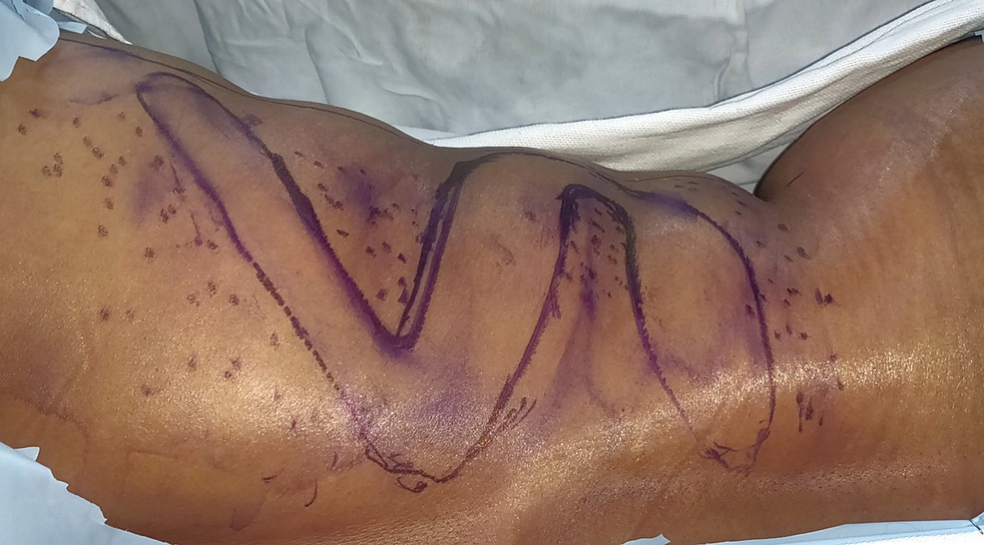
Latissimus dorsi flap marking with S-shaped skin paddle. The dotted area represents the extra subcutaneous fat to be harvested along with the skin paddle.

An LD myocutaneous flap was planned with an S-shaped skin paddle (Figure 2).

**Figure 3:**
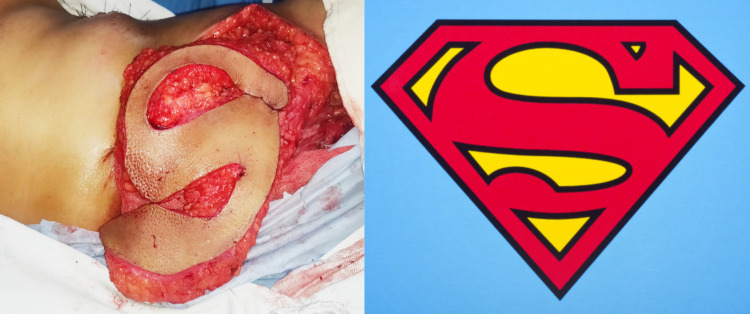
Harvested myocutaneous Latissimus dorsi flap with subcutaneous fat around the skin paddle. The design resembled the iconic Superman logo from the DC comics.

The patient’s backroll with maximum laxity was present in the middle of the LD flap territory, going oblong, where the middle part of the S-shaped was planned and marked. Keeping this flap in the pinched position where primary closure can be achieved, superior and inferior limbs of the S-shape were planned, allowing primary closure of the skin on the donor site. The final incision was planned with around 5mm inside of the pinched mark all along the planned incision of the S-shaped flap. The flaps were elevated in the subcutaneous plane with skin and subcutaneous flap thickness of approximately 1 cm. The maximum amount of subcutaneous fat was preserved over the muscle to capture the maximum number of perforators that will supply the skin paddle and add to the bulk. The anterior border of LD muscle was identified, and muscle was elevated, and all intercostal perforators were ligated. The muscle with its overlying paddle was dissected free from all its underlying and distal attachments, permitting a free arc of rotation. The thoracodorsal pedicle was dissected carefully, and the branches to the serratus anterior muscle were ligated. The flap with skin paddle was then tunneled to the defect for sculpting and inset (Figure 3).

**Figure 4:**
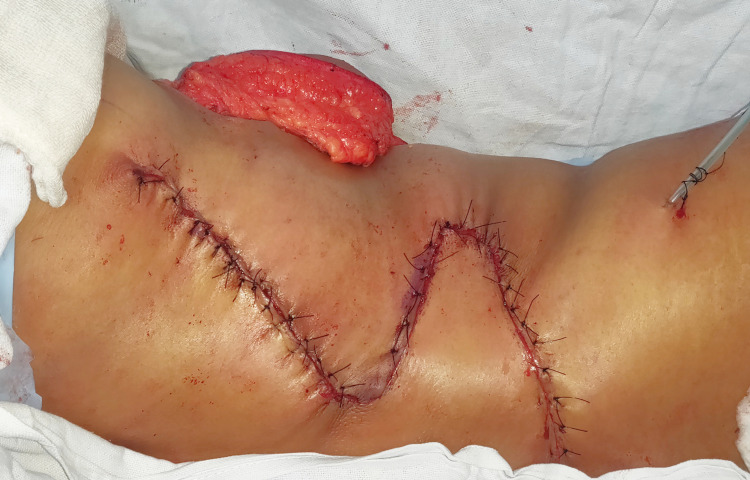
Primary closure of the donor site with tunneled Latissimus dorsi flap into the defect.

The donor site was closed primarily in three layers after placing a 16 Fr negative suction drain (Figure 4).

**Figure 5:**
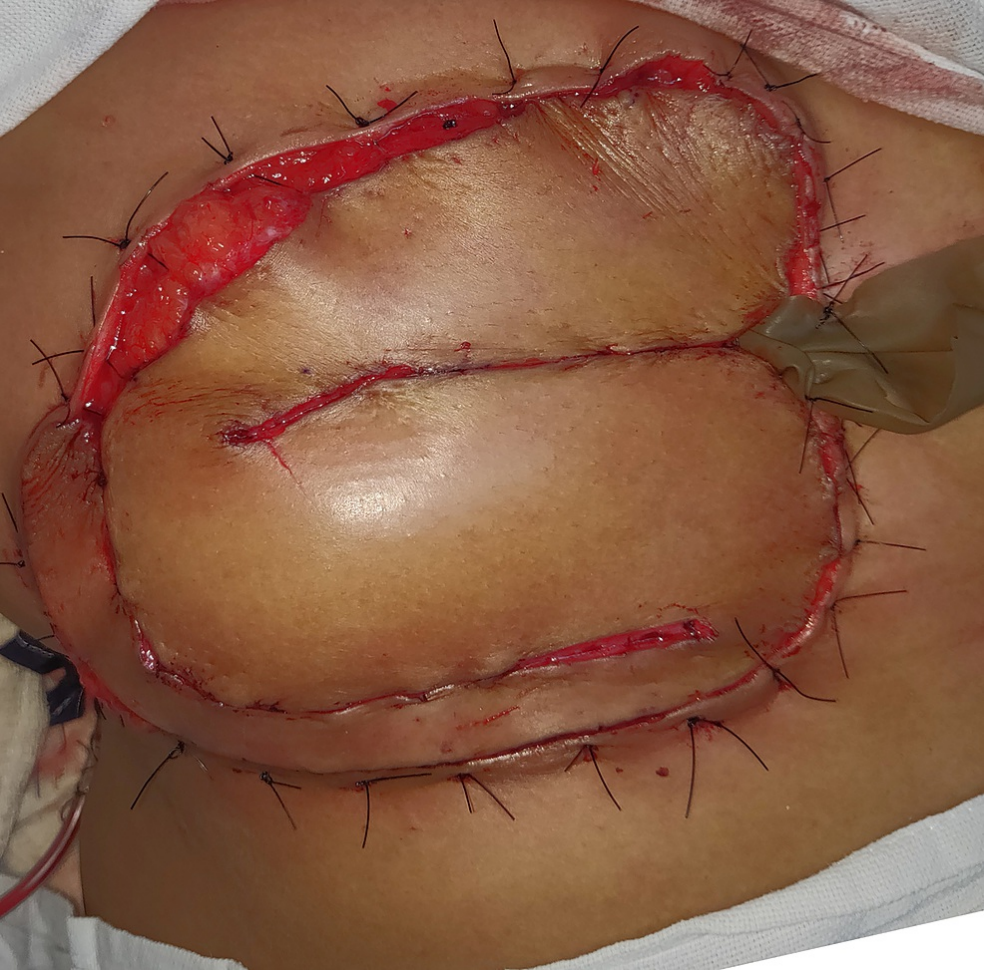
Inset of the flap into the chest wall defect.

After turning the patient back to a supine position, the horizontal limbs of S were sutured together. The combined length and width of the resultant paddle were sufficient to cover the defect. The flap inset was given to the surrounding skin using loose intermittent sutures (Figure 5).

**Figure 6:**
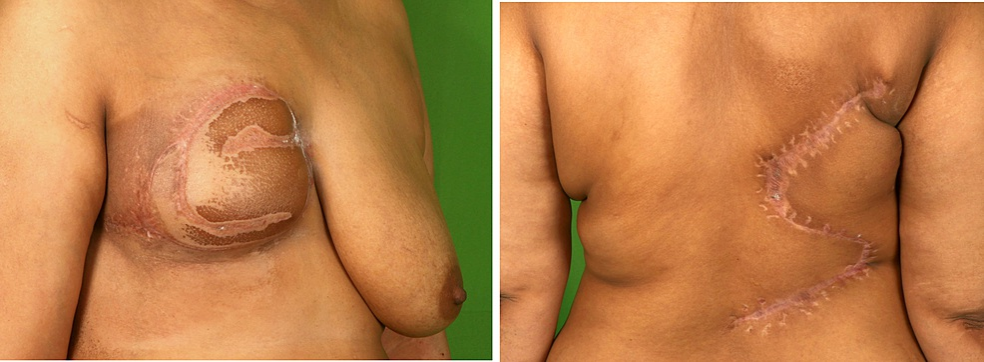
Six months post-operative follow-up pictures of the patient showing well-settled flap (left) with well-healed scar at the donor site (right).

The flap settled well, and the donor site healed without any complications. The patient received adjuvant radiotherapy after four weeks post-surgery (Figure 6).

## Discussion

Although several methods are described for post-mastectomy breast reconstruction, the LD flap remains the workhorse flap [8]. It is a robust, broad, and pliable flap with constant vascular anatomy. Its proximity to the breast allows a transfer of the muscle as a pedicle flap. The pedicled LD flap provides an excellent option for breast reconstruction in which a microvascular-free flap option is limited, or an abdominal flap cannot be done. It avoids a microvascular procedure and abdominal morbidity [9,10].

However, extensive post-mastectomy defects may require importing a larger amount of tissue to cover the defect and provide the volume. This may be achieved by using a single large free flap or multiple free flaps in combination. However, such procedures are associated with complicated microsurgical techniques, higher donor site morbidity, and failure rates [11,12]. One of the major drawbacks of using a pedicled LD flap is the limited width of the skin paddle that can be harvested allowing for the primary closure on the donor site. This limits its usage in extensive post-mastectomy defects. There have been various modifications proposed in the literature to overcome this limitation. Baumholtz et al. described a boomerang-shaped LD flap, which was later sculpted into a conically shaped tissue. It was then used to reconstruct the breast, providing more volume and better projection than the traditional LD flap [5]. The kiss LD flap described by Song et al. involves two semi-circular skin paddles at an angle in such a way that the straight lines of the two paddles intersect at one point. The skin paddles were later sutured together to form a complete circle and cover the defect. The donor site was closed primarily [6]. The fleur-de-lis pattern described by Ciudad et al. for extensive soft tissue defects utilizes a modified skin paddle with medial and lateral extensions just above the posterior superior iliac crest [7].

We believe that the S-shape of the skin paddle is unique in several ways. A large amount of skin and soft tissue can be harvested. The redundant tissue over both the upper and lower back can be utilized. The donor site scar is curvilinear or zig-zag and less prone to contracture. There is no scar junction or a meeting point of several scars (unlike in other designs) prone to dehiscence and stretch. The resultant shape of the skin paddle resembled a famous icon from the yesteryear DC comics, hence the name Superman flap. 

One of the major limitations of this technique is the inability to provide sufficient volume for breast projection. The priority in this case, however, was to cover the defect to start early radiotherapy. Adequate projection can be achieved with the help of an implant at a later date. Another major concern with the design is related to the vascularity of the distal-most skin paddle. While planning, one must ensure that it lies within the muscular territory of the flap and harvest as much as subcutaneous fat and the skin paddle to safeguard the perforators. The present technique was performed only in a single case; a larger series is required for it to become a standard of care.

## Conclusions

Versatility in designing the skin paddle of the LD flap permits the reconstruction of extensive mastectomy defects with low donor site morbidity. A novel design of skin paddle of LD flap has been devised and presented by the authors.
